# Nucleologenesis and embryonic genome activation are defective in interspecies cloned embryos between bovine ooplasm and *rhesus *monkey somatic cells

**DOI:** 10.1186/1471-213X-9-44

**Published:** 2009-07-28

**Authors:** Bong-Seok Song, Sang-Hee Lee, Sun-Uk Kim, Ji-Su Kim, Jung Sun Park, Cheol-Hee Kim, Kyu-Tae Chang, Yong-Mahn Han, Kyung-Kwang Lee, Dong-Seok Lee, Deog-Bon Koo

**Affiliations:** 1 Development and Differentiation Research Center, Korea Research Institute of Bioscience and Biotechnology (KRIBB), 111 Gwahangno, Yuseong-gu, Daejeon 305-806, Republic of Korea; 2 Department of Biological Sciences, Korea Advanced Institute of Science and Technology, Daejeon 305-701, Republic of Korea; 3 Aging Science Research Center, Korea Research Institute of Bioscience and Biotechnology (KRIBB), 111 Gwahangno, Yuseong-gu, Daejeon 305-806, Republic of Korea; 4 Department of Biology, Chungnam National University, Daejeon 305-764, Republic of Korea; 5 National Primate Research Center, Korea Research Institute of Bioscience and Biotechnology, 363-883, Republic of Korea; 6 School of Life Sciences & Biotechnology, College of Natural Sciences, Kyungpook National University, 1370 Sankyuk-dong, Buk-ku, Daegu 702-701, Republic of Korea; 7 Department of Biotechnology, Daegu University, Gyeongsan, Gyeongbuk 712-714, Republic of Korea

## Abstract

**Background:**

Interspecies somatic cell nuclear transfer (iSCNT) has been proposed as a tool to address basic developmental questions and to improve the feasibility of cell therapy. However, the low efficiency of iSCNT embryonic development is a crucial problem when compared to *in vitro *fertilization (IVF) and intraspecies SCNT. Thus, we examined the effect of donor cell species on the early development of SCNT embryos after reconstruction with bovine ooplasm.

**Results:**

No apparent difference in cleavage rate was found among IVF, monkey-bovine (MB)-iSCNT, and bovine-bovine (BB)-SCNT embryos. However, MB-iSCNT embryos failed to develop beyond the 8- or 16-cell stages and lacked expression of the genes involved in embryonic genome activation (EGA) at the 8-cell stage. From ultrastructural observations made during the peri-EGA period using transmission electron microscopy (TEM), we found that the nucleoli of MB-iSCNT embryos were morphologically abnormal or arrested at the primary stage of nucleologenesis. Consistent with the TEM analysis, nucleolar component proteins, such as upstream binding transcription factor, fibrillarin, nucleolin, and nucleophosmin, showed decreased expression and were structurally disorganized in MB-iSCNT embryos compared to IVF and BB-SCNT embryos, as revealed by real-time PCR and immunofluorescence confocal laser scanning microscopy, respectively.

**Conclusion:**

The down-regulation of housekeeping and imprinting genes, abnormal nucleolar morphology, and aberrant patterns of nucleolar proteins during EGA resulted in developmental failure in MB-iSCNT embryos. These results provide insight into the unresolved problems of early embryonic development in iSCNT embryos.

## Background

The derivation of human embryonic stem cells (hESCs) from somatic cell nuclear transfer (SCNT) blastocysts represents an innovative strategy for overcoming immune rejection during transplantation. However, autologous human therapeutic cloning using human donor cells and oocytes has been continuously faced with legal and moral quandaries. Thus, monkey primary cells and bovine oocytes have been used as alternative donor and recipient cells for SCNT, respectively. In addition, interspecies SCNT (iSCNT) shows promise as a technique for examining nucleocytoplasmic interactions [1], stem cells [2], and the cloning of endangered animals whose oocytes are difficult to obtain [3,4]. The most important application of iSCNT lies in its potential to facilitate the reprogramming of human somatic cells into embryonic stem cells, thus avoiding ethical issues associated with using human oocytes. Therefore, iSCNT may increase the feasibility of human therapeutic cloning by providing comprehensive information about a variety of developmental events.

Many iSCNT embryonic studies have used bovine oocytes or oocytes from a variety of other species, such as pigs, rats, sheep, and monkeys [1,5-8]. The bovine oocyte is one of the most popular recipient cytoplasts for iSCNT because of the large number of oocytes that can be retrieved and because the *in vitro *culture system is well established. Although bovine oocytes support development beyond the morula stage in dogs [9], humans [10] and monkeys [6], the poor developmental efficiency of iSCNT embryos remains a crucial problem when compared to *in vitro *fertilization (IVF) and intraspecies SCNT techniques. Some studies have reported that high rates of abnormal iSCNT development may result from aberrant gene expression [5,11,12] or epigenetic modification by DNA methylation [2].

Among mammals, embryonic genome activation (EGA) is the most critical event for viability during early development [13]. EGA occurs at the 2-cell stage in mice [14], at the 8- to 16-cell stage in humans [15] and bovines [16], and at the 6- to 8-cell stage in monkeys [17]. It requires the expression of the housekeeping genes HSP70 (cell cycle regulation), PGK1, and PDHA1 (glucose metabolism) [18], as well as imprinting genes such as NDN (a transcription activator) and XIST (X chromosome × inactivator) [19]. In addition, the transcription of ribosomal RNA (rRNA) serves as a marker for EGA and coincides with a dramatic increase in nucleolar gene activation in mice [20], bovines [21], and pigs [22], resulting from the formation of functional nucleoli. When the inactive nucleolus, or nucleolar precursor body (NPB), is transformed into an active nucleolus, it consists of the innermost fibrillar centers (FCs) surrounded by dense fibrillar components (DFCs), which are bordered by granular components (GCs) [23]. The FCs contains rDNA transcriptional enzymes, such as RNA polymerase I and upstream binding transcription factor (UBTF). The DFC, which delivers pre-mature rRNA to the GC, contains fibrillarin. The GC includes nucleophosmin and nucleolin, which are associated with the processing of premature rRNA [23]. The various nucleolar proteins must be localized in a specific nucleolar region for the formation of a functional nucleolus [24].

Impaired nucleologenesis often coincides with failed early development in SCNT embryos. The bovine ooplasm successfully supports initial nucleolar assembly in embryos cloned from bovine and porcine cells [25], whereas delayed nucleolar assembly and decreased fibrillarin content were found in mouse [26] and monkey embryos [27]. However, little or no data have been gathered regarding the reprogramming of a monkey donor genome within ooplasms of different species, especially that of the bovine, although the iSCNT strategy facilitates monkey embryogenesis studies and their subsequent applications.

The aim of the present investigation was to understand why monkey-bovine (MB)-iSCNT embryos have poor developmental rates to the blastocyst stage compared to IVF and bovine-bovine (BB)-SCNT) embryos. The objectives were to: 1) compare the developmental competence of IVF, BB-SCNT, and MB-iSCNT embryos to the blastocyst stage, 2) investigate the expression of housekeeping and imprinting genes, 3) compare nucleolar ultrastructure by transmission electron microscopy (TEM), and 4) compare the expression of nucleolar component proteins among IVF, BB-SCNT, and MB-iSCNT embryos by immunofluorescence confocal laser scanning microscopy and real-time polymerase chain reaction (PCR).

## Results

### Development of IVF, BB-SCNT, and MB-iSCNT embryos

**Figure 1:**
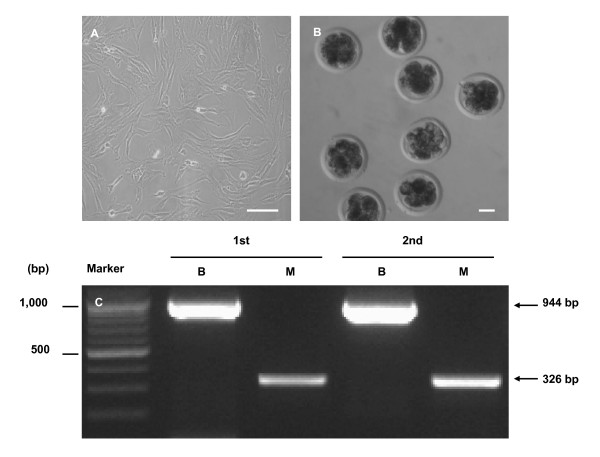
**Analysis of mitochondrial DNA (mtDNA) in monkey-bovine MB-iSCNT embryos on day 3**. Rhesus monkey ear skin fibroblasts (A), image of MB-iSCNT embryos at day 3 (B), and expression of mtDNA in MB-iSCNT embryos. Bar, 50 μm. B, bovine; M, rhesus monkey.

Rhesus monkey ear fibroblast cells (Figure 1A) were fused with enucleated bovine oocytes (MB-iSCNT) using the same fusion parameters used for bovine fibroblasts (BB-SCNT). IVF bovine embryos were also cultured to control for the effect of the nuclear transfer (NT) procedure. Eight-cell stage IVF, BB-SCNT, and MB-iSCNT embryos were observed (Figure 1B) after 3 days of culture. PCR-based mitochondrial DNA (mtDNA) analysis was used to confirm that 8-cell-stage MB-iSCNT embryos had fused. PCR amplification of D-loop mtDNA was performed using species-specific primers. Monkey and bovine mtDNA were detected in 8-cell iSCNTs (Figure 1C). No difference in cleavage rate was observed among MB-iSCNT (89.3 ± 2.7%, 99/110), IVF (86.3 ± 1.3, 101/117), or BB-SCNT embryos (85.3 ± 2.5%, 83/99; Table 1). The developmental rates of IVF and BB-SCNT embryos were 33.5 ± 2.8% and 28.2 ± 2.7%, respectively. However, the MB-iSCNT embryos did not develop into blastocysts (Table 1).

**Table 1:** Developmental capacity of embryos derived from IVF, BB-SCNT, and MB-iSCNT

		No. (%) of embryos developed to				
		
Group	No. of embryos cultured	Day 3				Day 7
		
		2-cell	4-cell	8- to 16-cell	Cleavage	Blastocyst
IVF	117	21 (17.9 ± 2.8)	37 (31.5 ± 2.7)	43 (36.9 ± 3.4)	101/117 (86.3 ± 1.3)	38 (33.5 ± 2.8)^a^
BB-SCNT	99	13 (14.1 ± 2.8)	13 (13.7 ± 2.1)	58 (57.4 ± 3.4)	83/99 (85.3 ± 2.5)	29 (28.2 ± 2.7)^a^
MB-iSCNT	110	12 (16.1 ± 4.1)	20 (22.3 ± 3.7)	67 (51.0 ± 5.1)	99/110 (89.3 ± 2.7)	0 (0)^b^

Cleavage and developmental rates are expressed as the mean ± standard error of the mean (SEM) from three independent experiments. The superscripts "a" and "b" indicate significant differences between experimental groups (*n *= 10, *P *< 0.05). BB: Bovine-Bovine; MB: Monkey-Bovine

### Down-expression of housekeeping and imprinting genes during EGA in MB-iSCNT embryos

**Figure 2:**
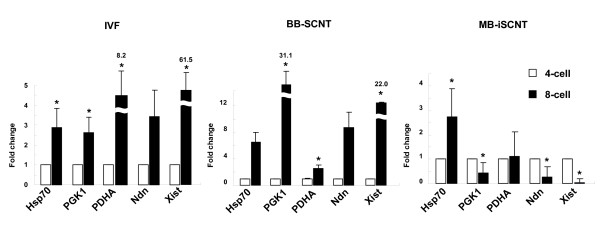
**Real-time PCR quantification of mRNA expression in IVF, BB-SCNT, and MB-iSCNT embryos**. Relative abundance of housekeeping and imprinting genes in IVF, BB-SCNT, and MB-iSCNT embryos between the 4- (before EGA) and 8-cell stages (after EGA). *Values are significantly different from the 4-cell control (*P *< 0.05).

We used the terminal transferase dUTP nick-end labeling (TUNEL) assay to examine the frequency of apoptotic cells in 8- to 16-cell-stage MB-iSCNT embryos on day 3.5; however, no difference was observed compared to the IVF and BB-SCNT embryos (data not shown). Next, we determined whether pre-EGA (4-cell stage) and post-EGA (8-cell stage) MB-iSCNT, IVF, and BB-SCNT embryos expressed the *HSP70, PDHA*, and *PGK1 *housekeeping genes and the *NDN *and *XIST *imprinting genes. Interestingly, there was an increase in the expression of the three housekeeping and imprinting genes in the 8-cell-stage IVF and BB-SCNT embryos compared to their respective 4-cell stages, but the MB-iSCNT embryos only expressed the *HSP70 *housekeeping gene during the same period (Figure 2). These results suggest that the failure of MB-iSCNT embryos to develop into blastocysts was due to the down-regulation of housekeeping and imprinting gene transcription during EGA.

### Abnormal nucleolar morphology during EGA in MB-iSCNT embryos

**Figure 3:**
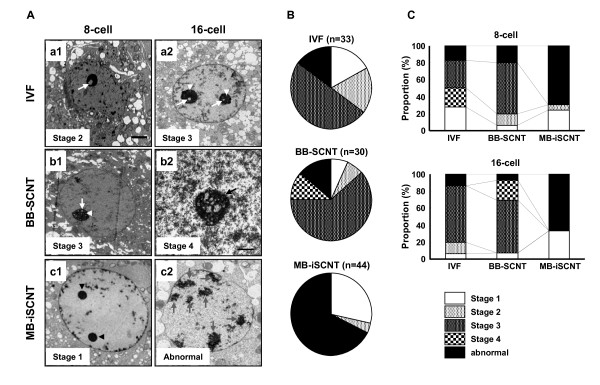
**TEM evaluation of nucleolar developmental stages in IVF, BB-SCNT, and MB-iSCNT embryos**. Primary vacuole (a1, white arrow) and both primary and secondary vacuoles (a2, white arrow head) in an IVF embryo. Both primary and secondary vacuoles (b1) and reticular nucleolus (b2, black arrow) in a BB-SCNT embryo. NPBs (c1, black arrow head) and abnormally structured nucleolus (c2, gray arrows) in an MB-iSCNT embryo. (B) Proportion of each nucleolar developmental stage in IVF, BB-SCNT, and MB-iSCNT embryos. (C) Proportion of each nucleolar development stage in both 8-cell and 16-cell stage embryos. Bar, 2 μm (A1, A2, B1, and C1); 1 μm (B2 and C2).

Given the abnormal housekeeping and imprinting gene expression during EGA in the MB-iSCNT embryos, we suspected abnormal transcription of the nucleus. Thus, we investigated the nucleolar ultrastructure of MB-iSCNT embryos during EGA and compared it to IVF and BB-SCNT embryos using TEM. We described the nucleologenic stages of the embryos according to Kopecny *et al*. [28] and evaluated the proportion of IVF, BB-SCNT, and MB-iSCNT embryos showing a given nucleolar stage during EGA. Control nuclei from IVF and BB-SCNT embryos contained NPBs with large and small vacuoles. Approximately 48% of the IVF and 60% of the BB-SCNT EGA-stage embryos were in stage 3 of nucleolar development (Figure 3B, top and middle, respectively), whereas few stage 3 nucleoli were observed among EGA-stage MB-iSCNT embryos (Figure 3A, a2, and 3A, b1). Interestingly, abnormal nucleolar structures, which were scattered near the nuclear membrane, were prevalent in MB-iSCNT embryos (68.2% of embryos; Figure 3A, c2, and 3B, bottom), indicating that they failed to develop normal nucleoli. Otherwise, the nuclear membrane, mitochondria, the Golgi apparatus, and other ultrastructural features were similar among all three types of embryos (data not shown).

Given that nucleologenesis is related to the embryonic developmental stage [29], we investigated the difference in the proportions of nucleolar-stage IVF, BB-SCNT, and MB-iSCNT embryos from early (8–11 cells) to late (12–16 cells) EGA. As shown in Figure 3C, there was a greater proportion of stage 3 nucleoli (IVF, 33.3%; BB-SCNT, 60.0%) than stage 2 (IVF, 22.2%: BB-SCNT 13.3%) or stage 1 (IVF, 27.7%; BB-SCNT, 6.6%) nucleoli among early-EGA IVF and BB-SCNT embryos. Abnormal nucleoli were found in 16.6% of IVF and 20.0% of BB-SCNT embryos. Similar results were observed for late-EGA IVF and BB-SCNT embryos. Interestingly, stage 4 (26.6%) nucleoli were only observed in late-EGA BB-SCNT embryos. Conversely, the MB-iSCNT embryos exhibited stage 1 (early, 24.1%; late 33.3%) and stage 2 (early, 6.8%; late 0%) nucleoli, but showed very large numbers of abnormal nucleoli (early, 68.9%; late, 66.6%). These results suggest that bovine enucleated oocytes have a limited capacity for supporting nucleologenesis in embryos produced via nuclear transfer from rhesus monkey somatic cells.

### Abnormal expression and disorganization of nucleolar component proteins in MB-iSCNT embryos

A variety of nucleolar component proteins must be expressed and localized in the appropriate nucleolar regions to form a functional nucleolus. However, our results indicate that failed early development in MB-iSCNT embryos is closely associated with abnormal nucleologenesis, as well as defects in EGA.

**Figure 4:**
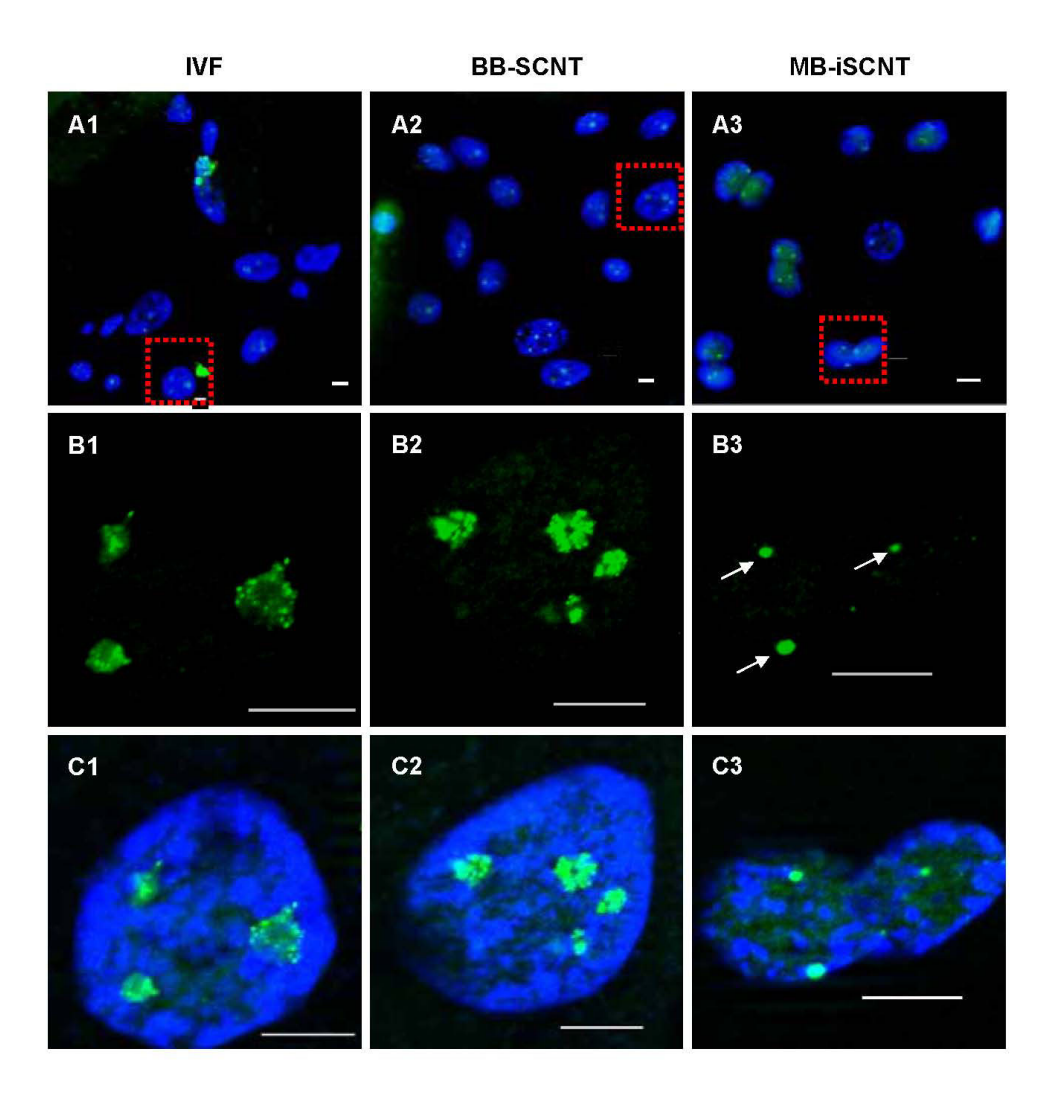
**Expression and localization of upstream binding transcription factor (UBTF) during embryonic genome activation (EGA)**. IVF (A1, B1, and C1) and BB-SCNT (A2, B2, and C2) embryos showed clusters of foci, whereas MB-iSCNT embryos showed only small foci (A3, B3, and C3). DNA was stained with DAPI. Arrow indicates small UBTF foci in an MB-iSCNT embryo. Bar, 10 μm.

**Figure 5:**
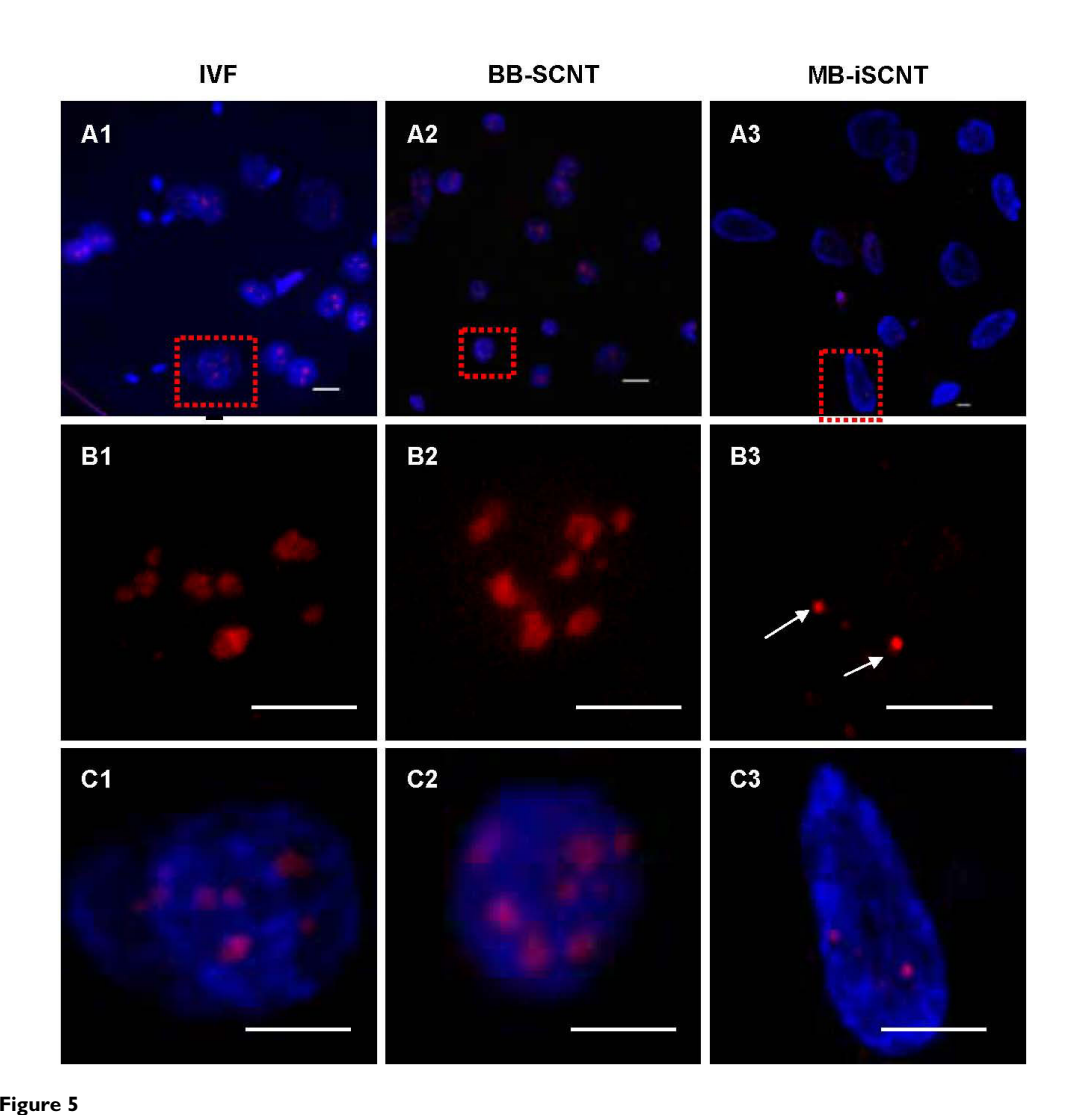
**Expression and localization of fibrillarin proteins during embryonic genome activation (EGA)**. IVF (A1, B1, and C1), BB-SCNT (A2, B2, and C2), and MB-iSCNT (A3, B3, and C3) embryos were immunostained with antibodies specific to fibrillarin. DNA was stained with DAPI. Arrow indicates small fibrillarin foci in an MB-iSCNT embryo. Bar, 10 μm.

**Figure 6:**
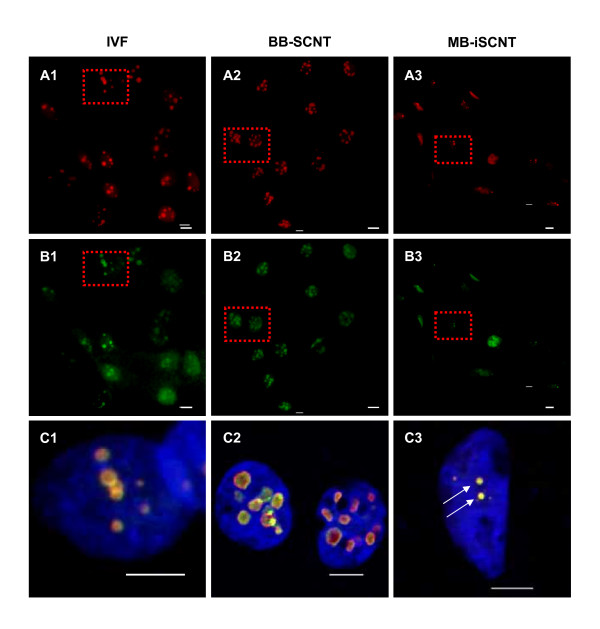
**Expression and localization of the nucleophosmin and nucleolin proteins during embryonic genome activation (EGA)**. IVF (A1, B1, and C1), BB-SCNT (A2, B2, and C2), and MB-iSCNT (A3, B3, and C3) embryos were immunostained with antibodies specific to nucleophosmin and nucleolin. DNA was stained with DAPI. Arrow indicates small foci of nucleophosmin and nucleolin staining in an MB-iSCNT embryo. Bar, 10 μm.

We investigated the expression and localization of nucleolar component proteins, including UBTF, fibrillarin, nucleophosmin, and nucleolin, in IVF, BB-SCNT, and MB-iSCNT embryos during EGA. UBTF is typically found in focal clusters in putative nucleoli [30]. Although UBTF was found in small focal clusters in interphase IVF and BB-SCNT cells (Figure 4C1 and 4C2), very few clusters were observed in MB-iSCNT embryos (Figure 4C3) and some nuclei did not express UBTF (data not shown). In previous studies, fibrillarin was localized in clusters of intensely labeled foci, and nucleolin and nucleophosmin were found in a shell-like structure that appeared as a ring-like image [29]. Unlike IVF and BB-SCNT embryos, fibrillarin was detected in some blastomeres derived from MB-iSCNT embryos (Figure 5B1, B2, and 5B3). However, fibrillarin expression was reduced in MB-iSCNT embryos and it was spot-shaped (Figure 5C3). Nucleophosmin and nucleolin were co-localized in the nucleolus in almost all IVF and BB-SCNT embryos and appeared as shell-like structures (Figure 6C1 and 6C2), whereas nucleophosmin and nucleolin expression levels were lower in MB-iSCNT embryos and were spot-shaped (Figure 6C3).

**Figure 7:**
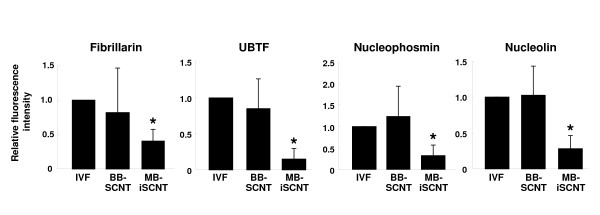
**Evaluation of nucleolar protein expression in embryos derived from IVF, BB-SCNT, and MB-iSCNT**. The expression of UBTF, fibrillarin, nucleophosmin, and nucleolin were relative to the mean expressed cells to the total nuclei in early (A) and late (B) EGA-stage embryos. Early EGA, 8- to 11-cell stage; late EGA, 12- to 16-cell stage. *Values are significantly different from the other groups (*P *< 0.05).

We quantified the expression of these proteins in individual blastomeres from IVF, BB-SCNT, and MB-iSCNT embryos in early (8–11 cells) and late EGA (12–16 cells). Although we observed significantly lower expression in early- and late-EGA stage MB-iSCNT blastomeres, we found similar protein expression rates in early and late IVF and BB-SCNT blastomeres (Figure 7A and 7B). Immunofluorescence confocal laser scanning microscopy results indicated that UBTF, fibrillarin, nucleophosmin, and nucleolin expression levels were significantly reduced in MB-iSCNT embryos compared to IVF and BB-SCNT embryos (Figures 4, 5 and 6). This may suggest that the abnormal expression and disorganization of nucleolar component proteins resulted in the formation of abnormal nucleoli in MB-iSCNT embryos.

## Discussion

Our results demonstrate that the abnormal expression and disorganization of nucleolar component proteins during EGA results in abnormal nucleolar structure, which leads to lower developmental rates in MB-iSCNT embryos. When the developmental rates of IVF, BB-SCNT, and MB-iSCNT embryos were examined, the cleavage rates of MB-iSCNT embryos, which received monkey fibroblast nuclei and bovine oocyte cytoplasm, were similar to those of IVF and BB-SCNT embryos; however, they failed to develop to the blastocyst stage (Table 1). These results suggest that MB-iSCNT embryos could not proceed through the transition from maternal to embryonic development. These findings also demonstrate that the ooplasm at the second metaphase, and not the nucleus, is principally responsible for early embryonic development prior to EGA, a finding consistent with results reported in iSCNT mice [12] and cats [31] generated using bovine enucleated oocytes.

The culture system is a very important factor affecting the efficiency of nuclear transfer. Non-human primate iSCNT embryos derived from donor human adult somatic cells and recipient bovine oocytes have been successfully cultured in IVC medium [1,3]. Similarly, Park *et al*. [12] reported that MB-iSCNT embryos developed to the 8-cell stage in CR1-aa medium, even though EGA occurs at the 2-cell stage in mouse embryos. Therefore, to identify whether the limited developmental competency of the MB-iSCNT embryos described here is related to the type of culture medium, we cultured the embryos using several well-known culture media, including CR1-aa, IVC-1, IVC-3, G-1, G-2, complete early cleavage, and complete blastocyst medium. However, no culture medium was capable of supporting the early embryonic development of MB-iSCNT embryos (see Additional file 1). These results suggest that the developmental competency of iSCNT embryos may be more dependent upon the donor species than the recipient species or the culture medium used.

A recent study showed that 20% of MB-iSCNT embryos exhibited DNA damage compared to SCNTs that were parthenogenetically activated (PA) on day 7 [8]. However, in the present study, the TUNEL assay revealed no difference in the frequency of apoptotic cells among IVF, BB-SCNT, and MB-iSCNT embryos on day 3 (data not shown). Therefore, it appears that developmental failure was not due to apoptosis. These results led us to hypothesize that the inability to progress through EGA was responsible for developmental failure in MB-iSCNT embryos. EGA is associated with transcriptional activation [32] and its timing is species-specific [14-16]. A failure to regulate the timing of activation or the types of genes expressed can lead to developmental arrest during preimplantation [13], and previous studies have shown that MB-iSCNT embryos undergo developmental arrest due to failed gene expression at the appropriate time [12]. In agreement with previous studies, we found an increase in the expression of *HSP70, PGK1, PDHA, Ndn*, and *Xist *in 8-cell-stage IVF and BB-SCNT embryos (Figure 2) [18,33]; in contrast, *PDHA, PGK1*, *Ndn*, and *Xist *expression decreased in MB-iSCNT embryos, indicating that blastocyst development did not occur in the 8-cell stage MB-iSCNT embryos due to a failure to complete EGA.

It is generally accepted that the transcriptional activity of the nucleus is associated with a change from a compact nucleolus (stages 1 and 2) to a functional nucleolus (stages 3 and 4), and that greatly increased transcription is indicative of EGA [34]. We observed stage 1 and 2 nucleoli during early EGA in MB-iSCNT embryos, but stage 3 nucleoli were sparsely observed in early or late EGA; furthermore, these embryos showed irregularly shaped NPBs (Figure 3). This finding coincided with those observed when transcription is inhibited by alpha-amanitin, which causes the disintegration of NPBs and embryonic developmental arrest [35]. Conversely, a greater proportion of stage 3 nucleoli were also observed in early-EGA BB-SCNT embryos than in early-EGA IVF embryos, and stage 4 nucleoli were only observed in late-EGA BB-SCNTs. We assumed that BB-SCNTs developed to the nucleolar stage at a faster rate than the IVF embryos, as in previous studies [36]. In addition, previous studies have reported that the nucleoli in late-EGA, 8-cell-stage IVF-derived embryos have nucleolar vacuoles, whereas 4-cell stage BB-SCNT embryos display both small and large vacuoles [21,37]. Interestingly, we previously observed that normal blastocyst development in goat-bovine iSCNT embryos was associated with functional changes in nucleoli (unpublished data), and development was similar to that of goat-goat SCNT embryos [34]. From an ultrastructural viewpoint, it is clear that MB-iSCNT embryos were unable to form a functional nucleolus during EGA. These results suggest that functional nucleoli are critical for the normal development of iSCNT embryos and that successful development may be donor species-dependant.

We demonstrated that developmental arrest in MB-iSCNT embryos may be caused by aberrant expression of nucleolar proteins during early embryonic development. Numerous additional studies have shown that abnormal nucleologenesis is observed during the development of iSCNT embryos [34]. However, until now, no direct evidence was available as to whether this nucleolar defect is a cause or a result of developmental failure in iSCNT embryos.

It was recently suggested that the aberrant expression of nucleolar proteins causes abnormal nucleologenesis in SCNT embryos [37], which leads to failed EGA and developmental arrest [35]. In particular, the expression of nucleolar proteins is frequently dependent upon epigenetic modifications such as genomic methylation. We attempted to determine whether the reduced nucleolar protein expression observed in MB-iSCNT embryos is due to epigenetic modification. However, no difference was observed in the methyl profiles of CpG islands in fibrillarin promoters between monkey donor cells and MB-iSCNT embryos (data not shown). Given these findings, we propose two possible hypotheses or mechanisms regarding the reduced expression of nucleolar proteins such as fibrillarin. One possible explanation is differences in histone modification, which were not examined in this study. Alternatively, it is possible that the bovine oocyte-derived transcriptional machinery was unable to express the monkey fibrillarin gene. For example, successful embryonic or full-term development of iSCNT embryos may depend on the accurate spatio-temporal action of donor cell species-specific transcription factors. To investigate these hypotheses, we are currently developing rescue strategies, including a lentiviral vector system expressing same-species transcription factors.

A functioning nucleolus requires numerous components [24,38], but the location of these components is not completely understood [23]. It is, however, well known that various nucleolar component proteins such as UBTF, fibrillarin, nucleophosmin, and nucleolin must be expressed and localized in the appropriate nucleolar region to form a functional nucleolus. UBTF is detected in first-cell cycle zygotic embryos, it is localized to small spherical bodies, and it is one of several transcription factors required for the binding of RNA polymerase I to rDNA in IVF embryos [39]. Fibrillarin is localized to the FCs and dense DFCs of nucleoli [29] and is associated with rRNA modification [40,41], ribosome assembly [42], nucleolar assembly [43], and early embryonic development [44], an indicator of EGA [45]. Nucleolin is a phosphorylated protein present in large amounts in nucleoli during active ribosomal biogenesis [46]; it is localized to DFCs and GCs of nucleoli [47] and plays essential roles in rDNA transcription, rRNA maturation, ribosome assembly, nucleocytoplasmic transport, and nucleologenesis [24,48]. Nucleophosmin is involved in the shuttling of proteins into the nucleolus [49]. As shown in Figures 3, 4, 5 and 6, the initial targeting of nucleolar proteins appears to be normal; however, the reduced expression of nucleolar protein seems to be associated with impairment in further nucleologenesis during EGA. Although we could not address the relationship between nucleolar protein expression and nucleologenesis, our findings suggest that impaired nucleologenesis is a major hurdle in iSCNT technology.

We examined the expression patterns of nucleolar component proteins and the localization of functional nucleoli in MB-iSCNT embryos. As in previous studies [21,37], UBTF and fibrillarin were displayed as clusters of small foci in the putative nucleolus of 8-cell-stage IVF and BB-SCNT embryos (Figures 4 and 5), and nucleolin and nucleophosmin appeared as ring-shaped structures at EGA (Figure 6); these results are consistent with a previous study [29]. However, in MB-iSCNT embryos, UBTF, fibrillarin, nucleolin, and nucleophosmin were sporadically detected as small foci (Figures 4, 5 and 6). These results indicate that the dysregulation of these nucleolar proteins may have led to nonfunctional nucleoli, which might be separated into DFCs and GCs, and the down-regulation of housekeeping and imprinting genes during EGA. As the result, MB-iSCNT embryos exhibited developmental arrest.

Finally, we observed significantly lower expression of these nucleolar proteins in individual MB-iSCNT blastomeres than in IVF or BB-SCNT blastomeres (Figure 7). These results suggest that it is important for MB-iSCNT embryos to continuously maintain the expression of nucleolar proteins during EGA. Our future studies will use an adenoviral vector to examine whether an increase in nucleolar component proteins during the first three cleavage cycles overcomes nucleolar dysfunction and allows development to reach the morula or blastocyst stage.

## Conclusion

MB-iSCNT embryos derived from donor monkey fibroblasts and bovine recipient oocytes did not develop to the blastocyst stage. We determined that this failure was caused by the down-regulation of EGA, and that it was not related to apoptosis. Instead, impaired nucleologenesis and aberrant nucleolar formation in MB-iSCNT embryos lead to developmental arrest. Abnormal expression and disorganization of the nucleolar component proteins also resulted in the down-regulation of EGA in MB-iSCNT embryos. These results provide insight into the early stages of development in iSCNT embryos, useful model for understanding nucleoar biology and will assist in the development of techniques to resolve these issues in iSCNT technology and spur the development of further applications.

## Methods

### Chemicals

Chemicals were purchased from Sigma Chemical Co. (St. Louis, MO, USA) unless otherwise indicated.

### In vitro maturation (IVM) and IVF

Bovine ovaries were collected from a local slaughterhouse and transported to the laboratory in 0.9% saline at 25–30°C. Cumulus-oocyte complexes (COCs) were aspirated from follicles (2–6 mm in diameter) using a disposable 10-ml syringe with an 18-gauge needle. Aspirated COCs with at least three layers of compact cumulus cells and homogeneous cytoplasm were washed three times in TL-HEPES (1 mg/ml bovine serum albumin [BSA] and low-carbonate TALP [50]). Ten oocytes were matured in 50 μl of the *in vitro *maturation medium in a 60-mm dish (Nunc, Roskilde, Denmark) under mineral oil for 20–22 h at 38.5°C under an atmosphere of 5% CO_2 _in air. The medium used for oocyte maturation was TCM-199 (Gibco-BRL, Grand Island, NY, USA) supplemented with 10% (v/v) fetal bovine serum (FBS; Gibco-BRL), 10 IU/ml pregnant mare's serum gonadotropin (PMSG), 0.6 mM cysteine, 0.2 mM sodium pyruvate, and 1 μg/ml 17β-estradiol. Following IVM, 15 oocytes were fertilized in 50 μl of fertilization medium with frozen-thawed sperm at a concentration of 2 × 10^6 ^cells/ml. When sperm were added to the fertilization drops, 2 μg/ml heparin, 20 μM penicillamine, 10 μM hypotaurine, and 1 μM epinephrine (PHE) were also added. After 18 h, cumulus-enclosed oocytes were stripped using gentle pipetting and transferred to CR1-aa medium containing 0.3% BSA for *in vitro *culture [51].

### Culture of donor cells

All of the experimental and animal care protocols were conducted in accordance with the Korea Research Institute of Bioscience and Biotechnology (KRIBB) Guidelines for the Care and Use of Laboratory Animals. Cell culture and assessment procedures have been previously described [33]. We used bovine ear skin fibroblast (bESF) cells and monkey ear skin fibroblast (mESF) cells as donor cells for intra- and interspecies nuclear transfer, respectively. To prepare primary bESF and mESF cells, bovine and monkey ear skins were manually cut into small pieces of approximately 1 cm^2 ^and chopped with a surgical blade on a 100-mm culture dish. The chopped tissue was incubated in 10 ml of 0.25% (w/v) trypsin/3.65 mM EDTA solution (Gibco-BRL) at 37°C for 30 min. The trypsin was inactivated by adding an equal volume of growth medium (Dulbecco's modified Eagle's medium [DMEM, Gibco-BRL] supplemented with 10% FBS). After removal of cellular debris and undigested cell masses, the cells were re-suspended in growth medium, seeded into 100-mm cell culture dishes, and cultured at 38.5°C under 5% CO_2 _in air for approximately 2 weeks until confluent. The fibroblasts were passaged three times before use as a source of donor nuclei for intra- or interspecies nuclear transfer. Cells were frozen in DMEM with 10% FBS and 10% dimethylsulfoxide (DMSO) and stored in liquid nitrogen until use.

### Somatic cell nuclear transfer and in vitro culture (IVC)

SCNT was performed using bESF and mESF cells as donor cells. Donor cells were plated in six-well plates and cultured in DMEM (Gibco-BRL) with 10% FBS until confluent. Donor cells were washed with phosphate-buffered saline (PBS, Gibco-BRL), digested with 0.25% trypsin-EDTA for 3 min, and then washed with DMEM containing 10% FBS. The cells were centrifuged at 150 × *g *for 2 min and re-suspended in PBS. Mature oocytes were enucleated with a glass pipette by aspirating the first polar body and MII plate of the partial cytoplasm in TL-HEPES containing 7.5 μg/ml cytochalasin B. The nuclei were stained with Hoechst-33342 and aspirated cytoplasm was viewed under ultraviolet light to confirm the removal of nuclei. A single cell was injected into the peri-vitelline space of the enucleated oocyte cytoplast. The reconstructed embryos were fused using a fusion chamber with two stainless steel electrodes (1 mm apart) in a fusion medium consisting of 0.3 M mannitol, 0.5 mM HEPES, 0.3% BSA, 0.1 mM CaCl_2_, and 0.1 mM MgCl_2_. A single direct current pulse of 1.6 kV/cm for 20 μs was applied using an Electro Cell Manipulator 2001 (BTX, San Diego, CA, USA). The fused embryos were activated using a modification of a previously described method [52]. Two hours after electrofusion, the fused embryos were activated with 5 μM ionomycin for 5 min; they were then treated with 2.5 mM 6-dimethyl-aminopurine (6-DMAP) in CR1-aa medium containing 0.3% BSA and incubated at 38.5°C under 5% CO_2 _in air for 4 h. *In vitro*-fertilized reconstructed embryos were transferred to CR1-aa medium containing 0.3% BSA and incubated at 38.5°C under 5% CO_2 _in air for 3 d. On day 3 of culture, the cleaved embryos were collected and the cleavage rate was evaluated. The embryos were then transferred to CR1-aa containing 10% FBS and cultured for an additional 4 days (evaluation of developmental rates at this time).

### Preparation of medium and culture of MB-iSCNT embryos

Some of the iSCNT embryos were cultured in CR1-aa containing 0.3% BSA and 10% FBS, whereas other reconstructed iSCNT embryos were cultured in the media indicated below. The following *in vitro *culture media were used only for MB-iSCNT embryo culture: IVC-1 and IVC-2 (IVF media series, InVitrocare Inc., Frederick, MD) supplemented with 10% human serum albumin; G-1 and G-2 media (Vitrolife AB, Kungsbacka, Sweden) containing human serum albumin; and complete early cleavage and complete blastocyst medium (Irvine Scientific, Santa Ana, CA) supplemented with 10% serum substitute supplement (SSS). Each medium was used in one of two steps in the culture process (early embryo development or blastocyst formation). We used the early development medium to support cleavage of MB-iSCNT embryos for 3 days and the blastocyst formation medium to support the already cleaved MB-iSCNT embryos for 4 days.

### Analysis of species-specific mtDNA

Amplification of D-loop mtDNA was used to confirm the fusion of MB-iSCNT embryos. We analyzed each 8-cell MB-iSCNT embryo via PCR using specific primers for monkey and bovine mtDNA. The primer sequences for each mtDNA gene were: monkey, (GenBank™ accession number, AY612638), 5'-TAT TGC ATA AGC TTC ATA AAT AAC TCT AGC-3' (sense), 5'-TTA TTT AAT AGA TAT GTG CTA TGT CCG ATG-3' (antisense); bovine (GenBank™ accession number, NC006853), 5'-AAA TGT AAA ACG ACG ACG GCC AGT AAT CCC AAT AAC TCA ACA C-3' (sense), 5'-AAA CAG GAA ACA GCT ATG ACC ACT CAT CTA GGC ATT TTC-3' (antisense). PCR was conducted with an initial step of 94°C for 10 min and 30 cycles of 94°C for 40 s, 60°C for 40 s, and 72°C for 45 s using the primer for the D-loop region of monkey mtDNA; when using the primer for the D-loop region of bovine mtDNA, PCR was performed with an initial step of 94°C for 10 min and 30 cycles of 94°C for 40 s, 55°C for 40 s, and 72°C for 45 s. The final 326- and 944-bp products were detected by agarose gel electrophoresis.

### Evaluation of zygotic gene expression in 4- and 8-cell embryos

We isolated the mRNA of 4- and 8-cell IVF, BB-SCNT, and MB-iSCNT embryos. Poly(A) mRNAs were extracted using a Dynabeads mRNA Direct kit (DYNAL), according to the manufacturer's instructions. After thawing, the samples were lysed in 300 μl of lysis/binding buffer (DYNAL) at room temperature for 10 min. Dynabeads oligo(dT) 25 (10 μl) were added to each sample. The beads were hybridized for 5 min and separated from the binding buffer using a Dynal magnetic bar. The poly(A) mRNAs and beads were washed in buffers A and B (DYNAL) and separated by adding 11 μl of diethylpyrocarbonate (DEPC)-treated water. The poly(A) mRNAs were reverse-transcribed in a total volume of 20 μl containing 500 μg/ml oligo(dT) primer, 10× PCR buffer, 20 IU RNase inhibitor, 200 U SuperScript II Reverse Transcriptase (Invitrogen, Madrid, Spain), 15 mM MgCl_2_, and 1 μl of dNTP mix (10 mM each). The secondary RNA structure was denatured at 65°C for 5 min; then, the cDNA was maintained at room temperature for 10 min and at 42°C for 60 min to allow reverse transcription. The reaction was terminated by heating at 70°C for 15 min. The inactivated cDNA was used as a template for PCR amplification. We used a previously described primer to detect monkey transcripts in the IVF, BB-SCNT, and MB-iSCNT embryos [18] and designed a bovine primer using the Primer3 program . An ABI 7500 Fast Real Time PCR System (Applied Biosystems, Inc., Foster City, CA, USA) and SyberGreen PCR Core reagents (Applied Biosystems) were used for RT-PCR. The primers are listed in Table 2. The *Hprt1 *gene level was used as the endogenous reference for each group.

**Table 2:** Primer sequences for real-time PCR analysis

Gene	Species	Sequence	GenBank accession no.	Size (bp)
HSP70	B	F:CCAGAGGAGGTGTCATCCAT R:GGGTGCTGGAAGAGAGAGTG	NM_174345	490
	M	F:TATTGGAGCCAGGCCTACAC R:GTCCGTAAAGGCGACATAGC	AF352832	168
PGK1	B	F:CTGCTGTTCCAAGCATCAAA R:GCACAAGCCTTCTCCACTTC	BC102308	202
	M	F:GTTGCACAGCATCTCAGCTC R:TCACTTGGTTTTAACAGGCAAA	AB125189	140
PDHA1	B	F:ATCCTCTGTCGTCCCCTTCT R:CTTAGACTGCAAGGCGATCC	XM_581602	187
	M	F:TGTCACAACAGTGCTCACCA R:CAAGCTTCCTGACCATCACA	AB083322	147
HPRT1	B	F:TGGCTCGAGATGTGATGAAG R:ACACTTCGAGGGGTCCTTTT	NM_001034035	370
	M	F:TTATACCACCGTGTGTTAGAAAAG R:ACACTACTAAAATAATTCCAGGACAGA	M31642	100
NDN	B	F:TCGCCAAGAATAGTGTGCTG R:TGAGTGGAAGAGCTGTGGTG	BC146188	110
	M	F:GACGAGGACGACCCGAAG R:ACTGGAGAGGTGGAATGTG	AB172756	149
XIST	B	F:TGCCACGCCTACAGTTAGTG R:GGGTTTTTCCCAGGTTGATT	BC146188	168
	M	F:TTACAGCAGGGGGTACTTGG R:AGGGAAGTGAGTGGGGTCTT	NR_001564	200

B: bovine; M: monkey

### Processing for TEM

Embryos at the 8- and 16-cell stages (84 h after insemination or after activation in the nuclear transfer group) were used for the TEM study. Cultured embryos were fixed with 3% glutaraldehyde in culture medium for 2 h at room temperature. They were then washed five times with 0.1 M cacodylate buffer containing 0.1% CaCl_2 _at 4°C and post-fixed for 2 h at 4°C with 1% OsO_4 _in 0.1 M cacodylate buffer (pH 7.2) containing 0.1% CaCl_2_. The embryos were rinsed with cold distilled water, transferred to micro-centrifuge tubes at 4°C, collected by centrifugation, embedded in 1% ultra-low gelling temperature agarose (type IX), slowly dehydrated in a graded ethanol series and propylene oxide at 4°C, and then finally embedded in Spurr's epoxy resin [53]. The resin polymerized after 36 h at 70°C and serial sections were cut with a diamond knife on an ULTRACUT ultramicrotome (Leica, Austria) and mounted on formvar-coated slot grids. Sections were stained with 4% uranyl acetate for 10 min and lead citrate [54] for 7 min. They were observed with a Tecnai G2 Spirit Twin transmission electron microscope (FEI Company, USA) and a JEM ARM 1300S high-voltage electron microscope (JEOL, Japan).

### Assessment of nucleolar developmental stages

Nucleolar structure was evaluated for developmental stages as described by Kopecny *et al*. [28], as follows: Stage 1, non-vacuolated NPB is an almost homogeneous fibrillar structure with densely packed fibrils; Stage 2, vacuolated NPB contains an eccentric center vacuole; Stage 3, nucleolus with secondary vacuoles; Stage 4, fully reticulated nucleolus in which the NPB has been transformed into a functional, rRNA synthesizing nucleolus. In the NPB, primary and secondary vacuoles were displayed.

### Assessment of nucleolar protein expression by immunocytochemistry

After assessing cleavage on day 3, the three types of embryos (8- to 16-cell stages) were fixed. The primary antibodies were: mouse monoclonal anti-UBTF (1:50, H00007343-M01; Abnova, Walnut, CA, USA), rabbit polyclonal anti-fibrillarin (1:50, sc-25397; Santa Cruz Biotechnology, Santa Cruz, CA, USA), mouse monoclonal anti-nucleophosmin (1:50, ab10530; Abcam, Cambridge, UK), rabbit polyclonal anti-nucleolin (1:50, ab16940; Abcam). The IVF, BB-SCNT, and MB-iSCNT embryos were fixed in 4% formaldehyde in PBS for 1 h at 4°C. The embryos and cells were washed for 30 min in PBS containing 0.1% Tween 20 (PBST) and then permeabilized for 1 h at room temperature in PBS-PVA containing 0.5% Triton X-100. The samples were treated with 2% BSA in PBST overnight at 4°C. The primary antibody was diluted 1:50 in PBST and co-incubated for 6 h at 4°C. After washing for 1 h, the samples were incubated for 30 min with Alexa 594 anti-rabbit IgG and FITC-conjugated anti-mouse IgM, and then washed for an additional hour. Samples were mounted on slides with mounting medium containing 1.5 μg/ml 4,6-diamidino-2-phenylindole (VECTASHIELD with DAPI; Vector Laboratories, Servion, Switzerland). The samples were viewed under a Zeiss AxioVert 200 M microscope with ApoTome (Zeiss, Oberkochen, Germany). The method to assess nucleolar protein expression was previously described [55]. We classified embryos as early stage (8–11 cells) or late stage (12–16 cells) and determined the proportion of nucleolar protein expressed in blastomeres to the total number of nuclei of embryos.

### Statistical analyses

All experiments were replicated more than three times. Data are presented as the mean ± standard error (SE) of the cultured oocytes. The data were analyzed via analysis of variance (ANOVA) followed by Duncan's multiple range test using the SAS software package (SAS Institute, Inc., Cary, NC, USA).*P *< 0.05 was considered statistically significant.

## Authors' contributions

BSS performed all experiments. SHL was responsible for analyzing TEM data. SUK, BSS, JSK, and JSP performed interspecies SCNT. CHK, KTC, YMH, and KKL provided animal care services (specifically monkey breeding), provided rhesus monkey ear skin fibroblasts, performed bovine oocyte culture, and participated in manuscript preparation. DSL and DBK initiated and directed this study, participated in the analysis of nucleolar protein expression via immunocytochemistry, and wrote most of the manuscript. All authors read and approved the final manuscript.

## Supplementary Material

Additional file 1 **Comparison of developmental capacity of iSCNT embryos by different culture media**. The data provided represent the development competence of iSCNT embryos by using different culture systems. Click here for file
