# Supplementary material for: Nucleologenesis and embryonic genome activation are defective in interspecies cloned embryos between bovine ooplasm and rhesus monkey somatic cells
Source: BMC Dev Biol. 2009 Jul 28;9:44. doi: 10.1186/1471-213X-9-44 (PMC2734572; doi:10.1186/1471-213X-9-44)
Supplement: Additional file 1 — Comparison of developmental capacity of iSCNT embryos by different culture media. The data provided represent the development competence of iSCNT embryos by using different culture systems. [file 1471-213X-9-44-S1.pdf]

Additional file 1. Comparison of developmental capacity of iSCNT embryos by different culture media

Group	No. of embryos cultured	No. (%) of embryos developed to				
		Day 3				Day 7
		2-cell	4-cell	8-16cell	Cleavage	Blastocyst rate
CR1-aa	57	3 (7.7±3.7)	5 (8.2±1.6)	45 (76.4±3.6)	90.1±2.3	-
IVC-1,3	55	5 (13.3±4.5)	7 (17.7±4.3)	35 (54.6±6.3)	86.9±2.7	-
G1,2	24	3 (12.5±0)	6 (21.9±3.6)	10 (43.8±3.0)	78.1±2.1	-
Complete 1,2	23	2 (10.0±3.8)	3 (12.7±2.0)	14 (59.6±3.4)	82.3±1.8	-
